# Machine Learning Identification of Nutrient Intake Variations across Age Groups in Metabolic Syndrome and Healthy Populations

**DOI:** 10.3390/nu16111659

**Published:** 2024-05-28

**Authors:** Chenglin Cai, Hongyu Li, Lijia Zhang, Junqi Li, Songqi Duan, Zhengfeng Fang, Cheng Li, Hong Chen, Metab Alharbi, Lin Ye, Yuntao Liu, Zhen Zeng

**Affiliations:** 1 College of Food Science, Sichuan Agricultural University, Yaan 625014, China; caichengl@163.com (C.C.);; 2 College of Information Engineering, Sichuan Agricultural University, Yaan 625014, China; 3 Department of Pharmacology and Toxicology, College of Pharmacy, King Saud University, P.O. Box 2455, Riyadh 11451, Saudi Arabia; 4 College of Animal Science and Technology, Sichuan Agricultural University, Chengdu 611130, China

**Keywords:** machine learning, XGBoost, Cox, metabolic syndrome, diet nutrition, age

## Abstract

This study undertakes a comprehensive examination of the intricate link between diet nutrition, age, and metabolic syndrome (MetS), utilizing advanced artificial intelligence methodologies. Data from the National Health and Nutrition Examination Survey (NHANES) spanning from 1999 to 2018 were meticulously analyzed using machine learning (ML) techniques, specifically extreme gradient boosting (XGBoost) and the proportional hazards model (COX). Using these analytic methods, we elucidated a significant correlation between age and MetS incidence and revealed the impact of age-specific dietary patterns on MetS. The study delineated how the consumption of certain dietary components, namely retinol, beta-cryptoxanthin, vitamin C, theobromine, caffeine, lycopene, and alcohol, variably affects MetS across different age demographics. Furthermore, it was revealed that identical nutritional intakes pose diverse pathogenic risks for MetS across varying age brackets, with substances such as cholesterol, caffeine, and theobromine exhibiting differential risks contingent on age. Importantly, this investigation succeeded in developing a predictive model of high accuracy, distinguishing individuals with MetS from healthy controls, thereby highlighting the potential for precision in dietary interventions and MetS management strategies tailored to specific age groups. These findings underscore the importance of age-specific nutritional guidance and lay the foundation for future research in this area.

## 1. Introduction

Metabolic Syndrome (MetS) represents a complex cluster of risk factors that significantly contribute to the development of cardiovascular diseases and type II diabetes. These factors include hypertension, dyslipidemia, hyperglycemia, and central obesity [1], which are becoming increasingly prevalent worldwide [2,3], alongside a parallel rise in obesity rates [4,5]. This trend presents a substantial challenge to global public health infrastructure. Given the intricate nature of MetS, crafting effective prevention and management strategies is crucial. Among these strategies, diet plays a pivotal role in both the development and management of MetS. A detailed exploration of dietary functions reveals its critical influence on metabolic health. Dietary components can directly affect the body’s metabolic processes, influencing blood pressure, lipid levels, glucose metabolism, and body fat distribution. By understanding and optimizing dietary intake, it is possible to mitigate the risk factors associated with MetS, offering a promising avenue for reducing its prevalence and the associated health burdens [6,7,8].

The traditional approach to diagnosing MetS involves the assessment of multiple clinical parameters [9,10], a process that is both time-consuming and costly, especially in large-scale population studies. Consequently, there is a pressing need for alternative strategies that can efficiently screen individuals at a high risk of MetS for further evaluation. In this context, the advent of machine learning (ML) offers promising avenues for the development of predictive models for MetS [11,12]. Recent advancements in computational capabilities have enabled the application of ML algorithms to predict MetS risk with high accuracy, using both conventional statistical analyses and novel ML methods. Significant contributions to this field include the work of Darko Ivanović, who developed an artificial neural network (ANN) model based on non-invasive, low-cost, and readily obtainable parameters such as sex, age, body mass index, waist-to-height ratio, and blood pressure. This model demonstrated high predictive values for MetS [13]. Similarly, Maria Trigka and colleagues employed various ML techniques to predict MetS, incorporating a range of factors, including demographic, socioeconomic, and clinical parameters, achieving an accuracy rate of 89.35% [11].

The aforementioned research predominantly targeted the prognostication of MetS within the general populace, devoid of delving into age-specific distinctions. In the contemporary era, the demographic shift towards an older population is palpable, rendering it imperative to question the applicability of findings from studies traditionally focused on younger cohorts or the general populace to an aging demographic [14]. With an eye on the aging population, our investigation endeavors to incorporate dietary habits into the predictive modeling framework to explore which specific nutrients may exert a considerable impact on people with MetS in different age groups. This approach not only addresses a critical gap in existing research but also underscores the potential for dietary interventions in the management and prevention of MetS [15,16].

As a result, in order to explore the relationship between MetS and age and nutrition and to find out which nutrition may have an effect on MetS, our research leverages the advanced capabilities of ML in conjunction with the proportional hazards model (Cox) to analyze data from ten cycles of the National Health and Nutrition Examination Survey (NHANES) from 1999 to 2018 [17,18]. Our study embarks on an exploration to meld dietary patterns with fundamental physical indicators to unveil predictive factors for MetS and optimal cardiometabolic health (OCH) across diverse age demographics. Following an intricate analysis of feature importance and through meticulous manual selection [19], we identified a series of predictive markers, encompassing both physical examination metrics and nutritional indicators. Subsequent investigations into these nutritional indicators revealed their significant correlations with MetS. This effort aims to deepen our comprehension of the pathogenesis of MetS, thereby establishing a solid groundwork for the development of evidence-based clinical prevention strategies and the creation of personalized treatment protocols.

## 2. Materials and Methods

### 2.1. Data Collection and Processing

**Figure 1:**
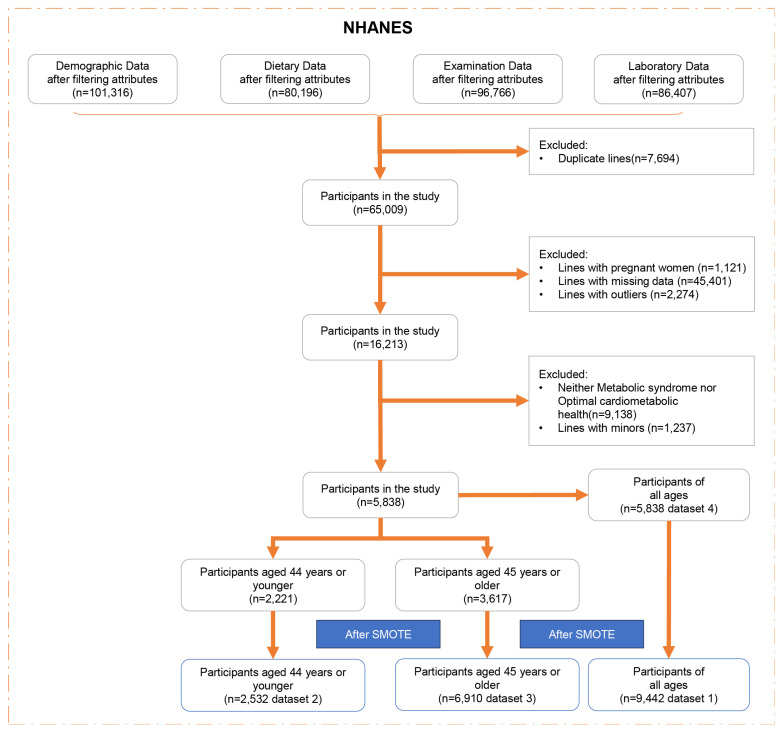
Participant selection and exclusion criteria flowchart. This schematic depicts the process of creating dataset 4 from four attribute-filtered datasets, consolidated into a table via sequence number (seqn), followed by the removal of duplicates, pregnant women, missing, and unreasonable data entries. Unreasonable data are defined as values exceeding five times the standard deviation above the attribute mean. Exclusions also include neither the metabolic syndrome nor the optimal cardiometabolic health population and minors, resulting in 5838 records, comprising 4721 MetS subjects and 1117 with OCH. Dataset 2 encompasses 2532 participants aged ≤44 years post-SMOTE processing. Dataset 3 includes 6910 participants aged ≥45 years, also post-SMOTE. Dataset 1 is derived from dataset 4, featuring 9442 entries post-SMOTE processing.

To enhance precision and mitigate sampling errors in our analysis, we amalgamated data spanning ten cycles (1999–2018) of the NHANES. NHANES, a cross-sectional, multistage survey, employs probabilistic sampling of the non-institutionalized civilian US population to effectively represent national demographics. Following data consolidation via “SEQN” linkage, we executed a series of preprocessing steps, including the removal of duplicate entries, exclusion of pregnant individuals, elimination of rows with missing data, excluding minors (age < 18), and identification and removal of outliers (defined as data points exceeding or falling below the mean by five times the standard deviation). Subsequent classification efforts partitioned the subjects into two distinct cohorts based on criteria for MetS and OCH, with the population neither meeting MetS criteria nor qualifying for OCH being excluded (Figure 1).

MetS was identified by the presence of any three or more of the following conditions [9]:

1. Waist circumference: >88 cm for women or >102 cm for men.
2. Triglycerides: >150 mg/dL.
3. HDL-C: <50 mg/dL for women or <40 mg/dL for men.
4. Blood pressure: systolic BP ≥ 130 mm Hg or diastolic BP ≥ 85 mmHg.
5. Fasting plasma glucose: ≥100 mg/dL.

OCH was defined by all of the following [20]:

1. Adiposity: BMI < 25 kg/m^2^ AND waist circumference < 88 cm for women or <102 cm for men.
2. Blood glucose: fasting plasma glucose < 100 mg/dL and HbA1c < 5.7%.
3. Blood lipids: total cholesterol to HDL ratio < 3.5:1.
4. Blood pressure: systolic BP < 120 mmHg, diastolic BP < 80 mmHg.

To address the issue of class imbalance observed across all datasets, the synthetic minority over-sampling technique (SMOTE) was utilized for the datasets. This technique is facilitated by the “imblearn version 0.12.2” Python package.

Following preprocessing, the dataset retained 5838 samples, which included 4721 subjects with MetS, 1117 exhibiting OCH, forming the basis for temporary dataset 1. A notable correlation between the incidence of MetS and age prompted division into two age groups (3.1 for details): the younger cohort (≤44 years) and middle-aged and elderly cohorts (≥45 years). This categorization yielded temporary datasets 2 and 3, respectively.

As a result, temporary datasets 1, 2, and 3 underwent processing via SMOTE to ensure equalized class representation across various age groups. Specifically, dataset 1 (n = 9442) featured a balanced compilation of basic data and nutritional indicators for all age groups; dataset 2 (n = 2532) catered to individuals aged 44 or younger; and dataset 3 (n = 6910) focused on those aged 45 or older, all benefitting from SMOTE’s balancing effect. In contrast, dataset 4 (n = 5838), which originated from temporary dataset 1, remained unprocessed by SMOTE. This dataset retained its original unbalanced nature but offered comprehensive coverage of basic data and nutritional indicators.

Each dataset comprised four physical examination indicators and sixty-three dietary indicators, including solely demographic information (sex, age), anthropometric information (height, weight), and nutritional intake (energy, carbohydrates, fiber, protein, cholesterol, etc.).

### 2.2. Model Selection

In the preliminary phase of developing the predictive model, we utilized logistic regression [21], random forest [22], extreme gradient boosting (XGBoost), and support vector classification (SVC). XGBoost, an advanced ensemble method, sequentially constructs decision trees, with each tree aimed at correcting the inaccuracies of its predecessors [17]. SVC, renowned for its versatility, discriminates between classes by identifying a hyperplane in the feature space that maximizes class separation [23]. To evaluate the predictive performance of each model under uniform training conditions, we set the max_iter for logistic regression at 500, and the n_estimators for both random forest and XGBoost at 500. Additionally, we applied min–max normalization to dataset 1 [24], partitioning it into the training set and the validation set following an 8:2 ratio.

### 2.3. Model Evaluation

In our analysis, we leveraged sensitivity, specificity, accuracy, the area under the receiver operating characteristic curve (AUC), and the area under the precision–recall curve (PR-AUC) to conduct a comprehensive evaluation of the models [25,26,27]. The calculations for some metrics are delineated as follows:

Accuracy = TP + TN/(TP + TN + FP + FN)(1)

Sensitivity = TP/(TP + FN)(2)

Specificity = TN/(TN + FP)(3)

The TP (true positive) refers to the proportion of patients accurately identified as having MetS. Conversely, FP (false positive) represents the fraction of individuals without MetS who are erroneously classified as having the condition. TN (true negative) denotes the proportion of healthy subjects correctly identified as not having MetS. Lastly, FN (true negative) pertains to the proportion of patients with MetS who are misclassified as healthy. All these metrics are normalized and thus range from 0 to 1, ensuring a standardized measure of diagnostic performance across varying sample sizes and study designs.

To assess the influence of nutritional indicators on MetS predictions, datasets 2 and 3 were subdivided into two categories: one containing solely demographic and anthropometric information (sex, age, height, weight) and the other encompassing both basic demographic information and nutritional indicators. To guarantee the methodological robustness of study, these subsets were allocated into training and testing groups following an 8:2 distribution ratio, with a randomized shuffling procedure to minimize bias.

### 2.4. Feature Verification and Screening

Then, we tried to identify which specific nutritional indicators exerted a pivotal influence. Datasets 2 and 3 were analyzed using the XGBoost algorithm. The model parameters were meticulously adjusted across a range of n_estimators (200, 500, 1000) and max_depth (3, 5, 7, 10) to refine the model’s performance. Notably, the disproportionate impact of height and weight on prediction outcomes necessitated a recalibration of feature importance weights. To mitigate this bias and ensure a balanced representation of nutritional intake indicators, the relative importance of height, weight, sex, and age was deliberately scaled down to three-twentieths of that assigned to other indicators after many experiments.

Through this methodological adjustment, the XGBoost model’s inherent feature selection mechanism, complemented by manual curation techniques [19], facilitated the identification of the 20 most critical nutritional indicators from a diverse range of parameters.

Then, datasets 2 and 3 were filtered to retain only the identified 20 nutritional and 4 demographic indicators. This curated dataset was then subjected to further analysis using the XGBoost model to establish a definitive ranking of feature importance. For this phase of the analysis, all features were assigned equal importance weights. However, given the direct correlation between height, weight, and the BMI—a critical determinant of MetS—the feature importance of height and weight was deliberately excluded from the final evaluation.

### 2.5. Risk Ratio Analysis

To evaluate the risk ratios associated with each indicator, we employed a Cox proportional hazards model for the analysis [18]. The Cox model was instantiated using the “lifelines version 0.28.0” package in Python. We used Cox model to evaluate the risk ratio of basic data indicators and nutrition indicators. Specifically, we conducted risk analyses for each nutritional intake with MetS. Given the absence of specific illness onset times for the MetS in our dataset, we standardized the illness time parameter across all instances to a value of 1.

A notable methodological adaptation was required to accommodate the Cox model’s constraint to numerical data inputs. Consequently, to integrate non-numeric data forms into the analysis, we employed a numeric encoding strategy. For example, educational attainment levels, serving as proxy indicators of socioeconomic status and lifestyle factors potentially influencing MetS risk, were quantified on an ordinal scale. Specifically, we assigned integer values in ascending order based on the level of education: “<HS grade” was encoded as 0, indicating high school not completed; “HS grade” was 1, representing high school completion; “Some college” was 2, denoting some college but no degree; and “College grade” was 3, corresponding to college graduation or higher levels of education.

### 2.6. Final Model Construction

In the concluding segment of our investigation, we directed our focus towards optimizing a predictive model that is universally applicable across diverse age cohorts. This endeavor necessitated the refinement of dataset 1, from which only four physical examination indicators and twenty nutritional indicators were retained.

The assessment of the final model’s efficacy entailed a structured comparison across various datasets, each representing distinct combinations of age groups and nutritional indicators. Specifically, the “All Age All Nutrition” dataset encapsulated the entirety of age demographics alongside a comprehensive array of nutritional indicators, as represented by dataset 1. In contrast, the “Some Age All Nutrition” dataset was confined to specific age brackets (either 44 years and younger or 45 years and older, as delineated in datasets 2 or 3) while still encompassing all nutritional indicators. The “Some Age Some Nutrition” dataset further narrowed the scope to include specific age groups in conjunction with a subset of indicators (24 evaluation indicators, derived from a filtration process applied to datasets 2 or 3). The “All Age Some Nutrition” dataset spanned all age groups but was limited to the same subset of indicators (24 evaluation indicators, obtained through the filtration of dataset 1).

## 3. Results

### 3.1. The Impact of Age on the Prevalence of MetS

These 4721 participants (69%) were classified as having MetS based on predefined criteria, while Table A1 presents a detailed comparison of characteristics between individuals with MetS and those with OCH. The MetS cohort exhibited a mean BMI that was 11.57 units higher and a mean age that was 31.68 years greater than their healthy counterparts.

**Figure 2:**
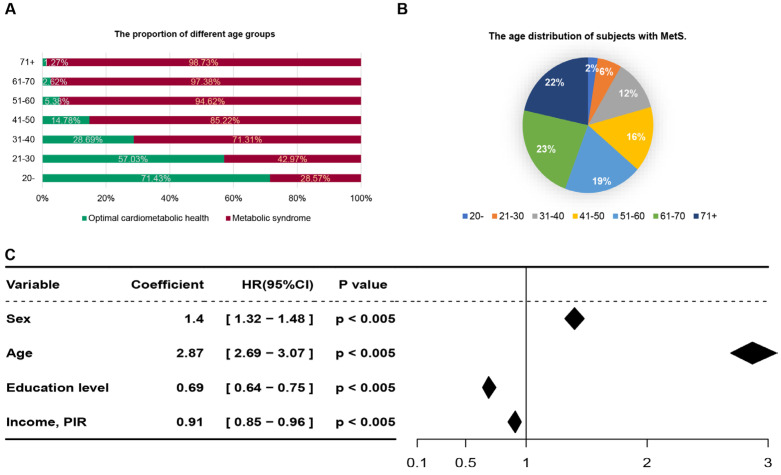
Original dataset (dataset 4) analysis. (**A**) A histogram analyzes the distribution of different age groups, along with the prevalence of OCH versus metabolic syndrome within these groups. (**B**) The age distribution of subjects with MetS. (**C**) Examination of the risk ratios of selected social factors (including sex, age, education level, race/ethnicity, and income PIR) to MetS in dataset 4. In the sex row, females are defined as 0, and males are defined as 1. In the age row, all ages 18 and older are included, those aged 44 and under are defined as 0 and those aged 45 and over are defined as 1. In the education level row, education is incremental; specifically, ‘<HS grad’ is defined as 0, ‘HS grad’ is defined as 1, ‘Some college/AA degree’ is defined as 2, and ‘College grad’ is defined as 3. Income, PIR was divided into two groups by their mean (2.47), where less than 2.47 was defined as 0 and greater than or equal to 2.47 was defined as 1. In the race/ethnicity column, we refer to the definitions in the NHANES dataset to place the following: ‘Mexican American’ is defined as 0, ‘Other Hispanic’ as 1, ‘Non-Hispanic White’ as 2, ‘Non-Hispanic Black’ as 3, and ‘Other Race’ as 4. The income PIR column contains the salary level of everyone in the dataset. A coefficient >1 suggests a positive correlation with metabolic syndrome risk, while <1 indicates a negative correlation. The diamond symbol represents the multivariable-adjusted hazard ratio, with width denoting the 95% CI.

Subsequent analysis focusing on age as a variable revealed a positive correlation between the prevalence of MetS and increasing age, alongside a corresponding decline in the incidence of OCH (Figure 2A). Specifically, within the MetS group, individuals aged 51 years and above constituted 64% of the total population diagnosed with the condition (Figure 2B). Additionally, we employed a Cox regression model to evaluate fundamental demographic information, including sex, age, education level, race/ethnicity, and income/poverty income ratio (PIR), identified age as a significant predictor with a coefficient value of 6.49, aligning with findings from prior research [28,29] (Figure 2C). In line with guidelines from the World Health Organization, the dataset was bifurcated into two age groups: ≤44 years and ≥45 years [30]. Post-application of the synthetic minority over-sampling technique (SMOTE) for data balancing, Table A2 and Table A3 further delineate the characteristics of individuals with MetS versus those in OCH within these age brackets. The analysis highlighted an average age discrepancy of 6.84 years and 8.52 years between the MetS and healthy groups in the younger cohort and middle-aged and elderly cohorts, respectively. These findings underscore the pivotal role of age in the development of MetS.

### 3.2. Evaluating Machine Learning Techniques for Predicting MetS Risk in Individuals

**Figure 3:**
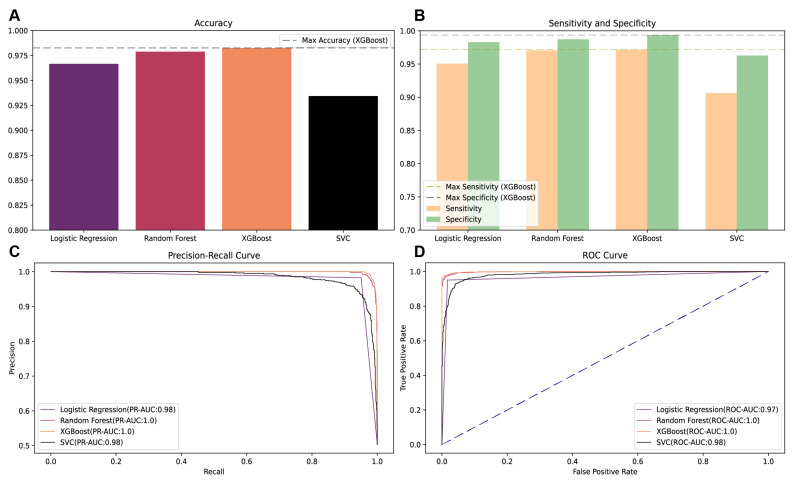
Model selection analysis. (**A**) Compares the accuracy of four models using dataset 1’s validation set. (**B**) Assesses the sensitivity and specificity of these models. (**C**) Shows the precision–recall curve. (**D**) Illustrates the ROC curve for model evaluation using dataset 1’s validation set.

To ascertain the most efficacious predictive model for MetS risk, we meticulously compared the performance of several ML algorithms, including logistic regression (LR), random forest (RF), XGBoost, and support vector classifier (SVC) on the validation dataset in dataset 1. The evaluation metrics indicate that the XGBoost model outperformed its counterparts, achieving the highest precision–recall area under the curve (PR-AUC) of 0.9985, surpassing the RF model’s PR-AUC of 0.9982 (Figure 3). In terms of sensitivity and accuracy, XGBoost demonstrated superior performance with a sensitivity of 97.1% and an accuracy rate of 98.2%, respectively. Additionally, it also attained the highest specificity score of 99.3%. Given its exemplary performance across multiple evaluation metrics, the XGBoost algorithm was selected for the development of the subsequent predictive model.

### 3.3. Investigating the Impact of Nutritional Intake on MetS

**Figure A2:**
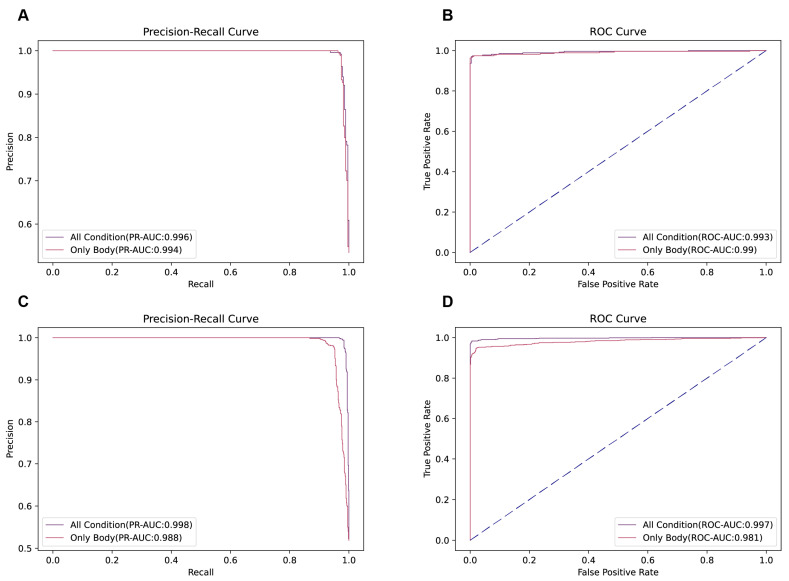
Age group dataset comparison. (**A**,**B**) Precision–recall and ROC curves for age group ≤44. (**C**,**D**) Precision–recall and ROC curves for age group ≥45.

To elucidate the influence of dietary habits on the risk of developing MetS, we partitioned the data according to age and whether to add nutritional indicators into four distinct datasets for analysis. The first and second datasets are both for people aged 44 years and below, and they differ in that the first dataset includes only demographic and anthropometric information (height, weight, sex, age), while the second dataset encompassed these basic data in addition to detailed records of nutritional intake. The third and fourth datasets are both 45 years and older, and they differ in the same way. Utilizing the XGBoost algorithm, we then trained and validated models using these datasets to assess the predictive relevance of dietary information on MetS across different age groups (Figure A2).

In the younger cohort, the dataset integrating nutritional intake alongside basic data exhibited a precision–recall area under the curve (PR-AUC) of 0.996, higher than the 0.994 PR-AUC observed for the dataset limited to basic data. Conversely, the area under the curve (AUC) for the receiver operating characteristic (ROC) analysis indicated a slight improvement with the inclusion of dietary information (0.993) compared to the basic data alone (0.990).

Conversely, for middle-aged and elderly cohorts, the addition of nutritional intake data markedly enhanced the model’s performance. This was evidenced by superior PR-AUC (0.998 vs. 0.988) and AUC (0.997 vs. 0.981) metrics for the dataset containing both basic and dietary information relative to the dataset restricted to basic data.

Our analysis substantiates the premise that nutritional intake possesses a discernible effect on the risk of MetS, with a more pronounced impact observed within middle-aged and elderly cohorts [31,32]. These findings underscore the importance of incorporating dietary data in predictive models to enhance their accuracy and reliability in identifying individuals at heightened risk of MetS, particularly among those aged 45 and above.

### 3.4. Feature Importance Analysis in MetS Risk Prediction

**Figure 4:**
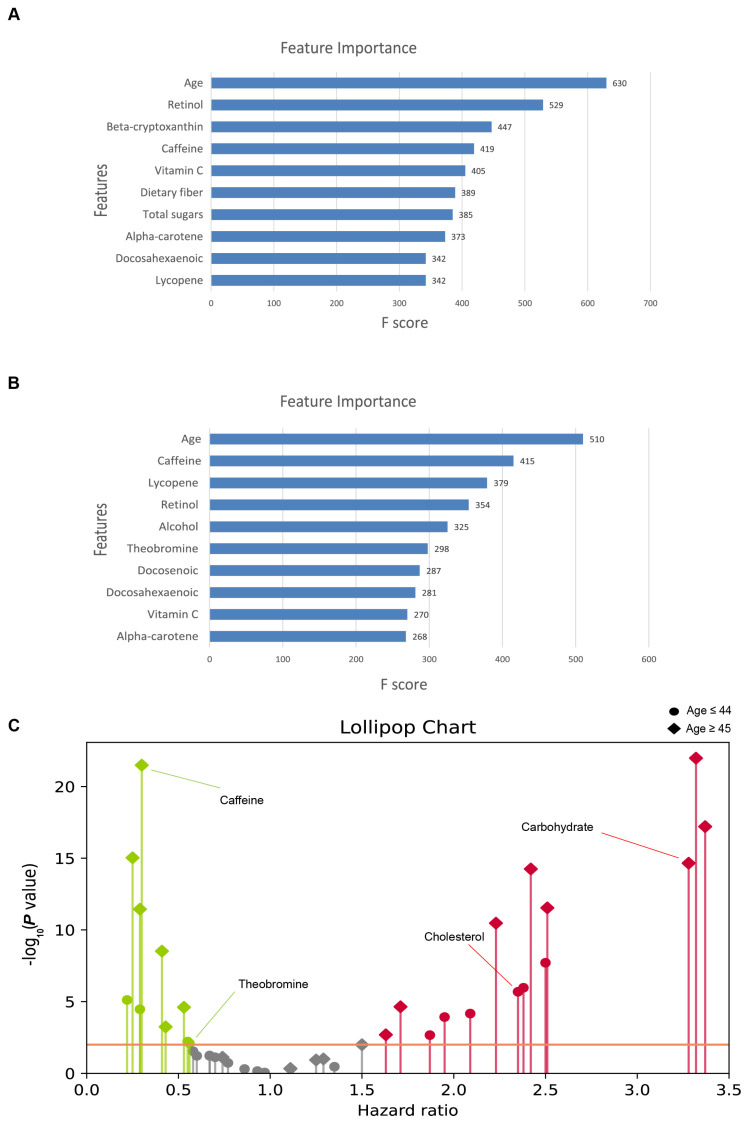
Dietary factors: feature importance and *p*-value distribution. (**A**) The top 10 feature importance in the XGBoost model for age ≤44. (**B**) The top 10 feature importance in the XGBoost model for age ≥45. (**C**) The *p*-value distribution from two-sided Wald tests, with the Y-axis showing the negative logarithm of each exposure’s *p*-value. The dotted red line indicates the *p*-value threshold of 0.01. Significant nutrients negatively associated with metabolic syndrome (HR < 1) are highlighted in green, and those with a positive association (HR > 1) are in red.

In our investigation into the determinants of MetS risk, we employed the XGBoost algorithm, leveraging a curated set of 24 features to elucidate their respective contributions to the prediction of MetS (Figure 4, Table A6 and Table A7). It is worth noting that BMI is one of the determinants of OCH, and it is not meaningful to analyze height and weight, which form BMI, so we removed height and weight from the final report.

For the younger cohort, the variables with the greatest significance were age, retinol, beta-cryptoxanthin, caffeine, and vitamin C, highlighting the important role of these factors in MetS prediction. Conversely, the feature importance ranking for middle-aged and elderly cohorts identified age, caffeine, lycopene, retinol, and alcohol as the top contributors, which may indicate variances in nutrient impacts with advancing age.

Consistently, age emerged as the leading factor in both age groups, reinforcing its pivotal role in MetS predisposition. The differential ranking of dietary components between the two age groups underscores the potential age-dependent effects of dietary intake on MetS risk. Remarkably, retinol maintained a high position in the feature importance hierarchy across both datasets, signifying its potential as a critical dietary factor in the risk assessment of MetS.

Furthermore, the analysis indicated a relatively minor role of sex in determining MetS risk, suggesting that the influence of age and dietary factors predominates over sex-specific effects in this context.

### 3.5. Nutritional Intake and Its Association with MetS

To elucidate the directional influence of dietary components on the risk of MetS, beyond merely their importance, we employed Cox proportional hazards models to assess the risk ratios associated with 20 specific nutritional intake features (Figure 4C). The results organize the nutritional exposures by their estimated hazard ratios (HRs), delineating those with HRs greater than 1.0 as promotive of MetS and those with HRs less than 1.0 as inhibitive. The distribution pattern revealed that significant associations predominantly clustered away from an HR of one, with exposures exhibiting small *p*-values dispersed at both extremes of the spectrum.

Our analysis identified 24 significant associations across datasets segmented by age (Table A4 and Table A5), with a *p*-value threshold set at <0.01 for proportionality assignments. In the younger cohort, cholesterol and total sugars were hypothesized to increase risk, whereas vitamin C, theobromine, and alpha-carotene were hypothesized to be protective. In contrast, for the middle-aged and older cohorts, variables such as energy, carbohydrates, and cholesterol were hypothesized to be associated with increased risk, whereas caffeine, theobromine, and alcohol were predicted to be protective.

Notably, alcohol intake revealed an inverse relationship with MetS risk (HR 0.29; 95% CI: 0.20–0.41; *p*-value < 4 × 10^−12^) despite relatively low consumption levels within the study population. This observation underscores the nuanced role of alcohol in metabolic health, warranting further investigation.

**Figure 5:**
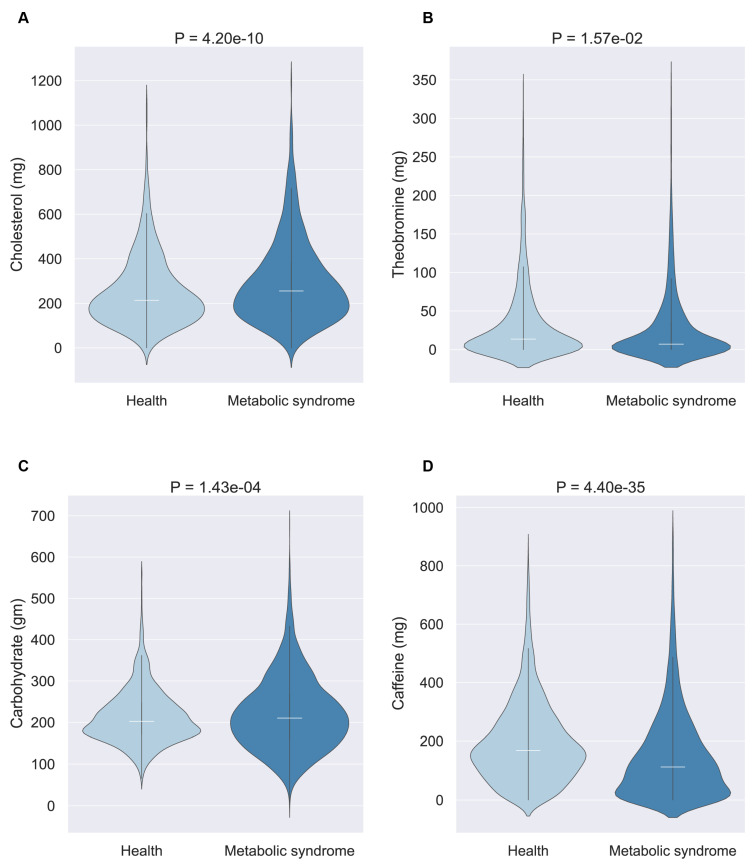
Significant nutritional elements by age group. The white horizontal line represents the average value. (**A**) Cholesterol as a possible metabolic syndrome-promoting nutrient in patients aged ≤44. (**B**) Theobromine as a possible metabolic syndrome-inhibiting nutrient in patients aged ≤44. (**C**) Carbohydrates as possible metabolic syndrome-promoting nutrients in patients aged ≥45. (**D**) Caffeine as a possible metabolic syndrome-inhibiting nutrient in patients aged ≥45.

Then, we selected four nutrients for an in-depth examination: theobromine and cholesterol (for the younger cohort) and caffeine and carbohydrates (for middle-aged and elderly cohorts). The violin diagram illustrates the consumption patterns of these nutrients between populations with and without MetS (Figure 5). This analysis revealed that beneficial nutrients were generally consumed in higher quantities by the healthy population, whereas harmful nutrients were more frequently consumed by individuals with MetS, suggesting a tangible impact of these selected nutrients on MetS development.

Furthermore, we found that cholesterol has a negative effect on people of all ages. Cholesterol presented a risk for the younger cohort (HR 2.35; 95% CI: 1.65–3.34; *p*-value < 3 × 10^−6^) and also presented a risk for middle-aged and elderly individuals (HR 2.23; 95% CI: 1.76–2.83; *p*-value < 4 × 10^−11^), which is in line with previous findings in the literature [33,34].

In contrast, the risk ratio of vitamin C in the younger cohort caught our attention, with a risk ratio of 0.55 representing the strong likelihood that it would have some inhibitory effect on MetS. This is also consistent with the findings of the previous article. In fact, the mechanism of action of vitamin C on MetS may be diverse and may be achieved by a combination of its antioxidant properties, protective effects against oxidative stress, anti-inflammatory properties, and other potential biological functions [35,36].

Differently, caffeine and theobromine exhibited more pronounced benefits for middle-aged and elderly cohorts, potentially due to their roles in addressing age-related health challenges, including cognitive decline, oxidative stress, and lipid metabolism disorders. The antioxidative properties of theobromine and the cardiovascular benefits associated with both caffeine and theobromine highlight the complex interplay between age, nutrient intake, and metabolic health [37,38,39].

### 3.6. Model Optimization for Predictive Analysis across Age Groups

In our endeavor to devise a predictive model capable of accurately forecasting MetS across a diverse age spectrum, we leveraged a dataset enriched with 24 meticulously selected features. This dataset encompassed the features above, including both demographic and nutritional intake indicators, tailored to enhance the predictive precision of our model.

**Figure A3:**
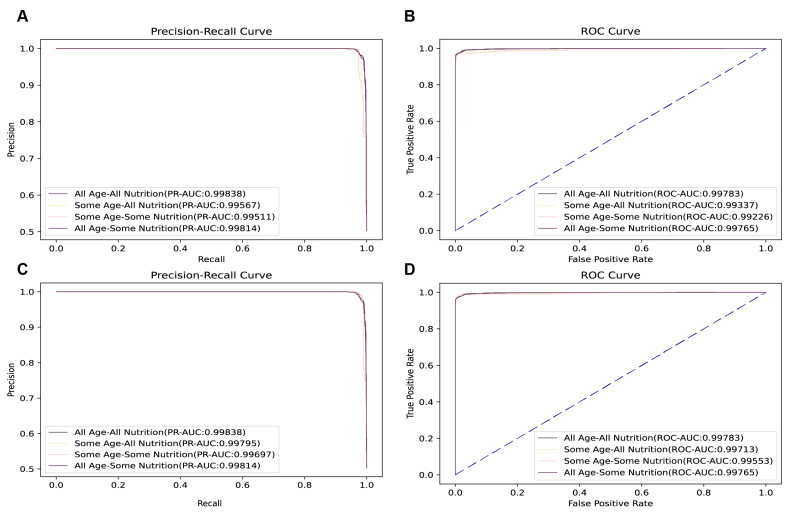
Model performance comparison across different datasets. It evaluates the effectiveness of models trained with various datasets: “All Age All Nutrition” utilizes a dataset with all nutritional indicators across all ages, “Some Age All Nutrition” uses a dataset with all nutritional indicators for specific age groups, “Some Age Some Nutrition” involves a dataset with selected nutritional indicators for specific ages, and “All Age Some Nutrition” employs a dataset with selected nutritional indicators across all ages. (**A**) A comparison of four models trained with different datasets for age ≤44. (**B**) An analysis of the sensitivity and specificity of models in (**A**). (**C**) A comparison of four models for age ≥45 trained with different datasets. (**D**) An evaluation of the sensitivity and specificity of models in (**C**).

Empirical evidence from our analyses clearly indicates that the “All Age All Nutrition” model is the pinnacle of predictive efficiency, with a ROC of 0.99783 (Figure A3), and that “All Age Some Nutrition” achieved the second performance in all ages. Compared to “All Age All Nutrition”, “All Age Some Nutrition” required 43 fewer metrics and had a decrease in PR-AUC and ROC of less than 0.1%. The superior performance of the model constructed by “All Age Some Nutrition” emphasizes its robustness in predicting age-specific demographics, further demonstrating its excellent sensitivity metrics. The comprehensive evaluation of its performance metrics confirms its overall efficacy and acceptability for a wide range of predictive applications.

## 4. Discussion

The exploration of the interplay between nutritional intake and MetS has been limited, prompting our investigation using advanced deep learning methodologies, particularly the XGBoost algorithm. This study aimed to predict MetS and identify the OCH population leveraging four physical examination indicators and twenty nutritional indicators. The dataset comprised 5838 samples from the NHANES, enhanced through synthetic minority over-sampling technique (SMOTE) to address class imbalance. The model achieved very good results, showing that it is very effective in distinguishing MetS.

Our analysis extends beyond traditional models by incorporating nutritional indicators alongside physical indicators, distinguishing between OCH and MetS populations. This approach contrasts with earlier studies by Darko Ivanović, Maria Trigka, and others, who predominantly focused on basic demographic and physical parameters, and through these parameters, they predict individuals with MetS in the population, while our study focuses on nutritional indicators alongside physical metrics. By studying the OCH and MetS populations, we have hypothesized a number of factors that have an impact on MetS. The differences in research may stem from different research purposes; our research focuses on theory, which is to identify factors that may have a significant impact on MetS.

In our investigation, we employed sophisticated ML techniques alongside univariate analyses of fundamental physiological markers and dietary intake indicators. This approach enabled us to elucidate previously obscure correlations between diet and health metrics. Our findings highlight the differential impact of age-specific dietary patterns on the prevalence of MetS. We discovered that the influence of certain dietary components, including retinol, beta-cryptoxanthin, caffeine, and vitamin C, was more pronounced in younger cohorts. Conversely, in middle-aged and elderly cohorts, the dietary effects of caffeine, lycopene, retinol, and alcohol were more marked. Subsequent investigations revealed that both young healthy cohorts and their middle-aged and elderly counterparts exhibited higher consumption levels of theobromine or caffeine. In contrast, an increased intake of cholesterol or carbohydrates was observed in young and middle-aged/elderly cohorts diagnosed with MetS.

Vitamin C intake, on the other hand, may be beneficial in younger populations, which is consistent with previous findings, and may be related to the anti-inflammatory properties of vitamin C. Individuals with MetS experience chronic systemic inflammation, and vitamin C has been shown to play a beneficial role in reducing the inflammatory response in the body [35]. In patients with MetS who were provided with a balanced diet and 500 mL/day of orange juice, an increase in vitamin C intake but a decrease in CRP and high-sensitivity C-reactive protein (hsCRP) levels were observed after three months of intervention [40].

Moreover, this investigation unveiled that different nutritional intakes may exert disparate pathogenic risks for MetS across various age groups. Notably, our study suggests that cholesterol intake may increase the risk of MetS in both younger and middle-aged and older cohorts. Conversely, the intake of caffeine and theobromine exhibits a possible beneficial effect in the middle-aged and elderly groups. This divergence may be attributed to age-related physiological transformations, including decelerated metabolism, alterations in sex hormone levels, cognitive function decline, augmented oxidative stress, and the dysregulation of lipid metabolism [41,42,43]. These findings contribute novel insights into the intricate relationship between diet and MetS, expanding our understanding beyond the existing literature.

Our findings elucidate that identical nutritional components may exert different effects across distinct age demographics. Such observations underscore the necessity of considering patient age when formulating nutritional recommendations for individuals with MetS. Concurrently, our research suggests that an increased intake of theobromine and caffeine, coupled with a reduced consumption of cholesterol and carbohydrates, may contribute to the mitigation of MetS symptoms. Moreover, the role of retinol within various age brackets emerged as a focal point of interest in our study. After consulting information, we found that retinol binds to retinol binding protein (RBP) in the blood and affects energy homeostasis and insulin response [44,45]. Moreover, retinol also has anti-inflammatory effects and has an impression of lipid metabolism [46,47,48], indicating that retinol may have a positive impact on MetS through multiple pathways.

Leveraging deep learning methodologies enables us to discern the significance and risk ratios associated with nutritional factors in the context of MetS. While conventional statistical approaches facilitate the identification of disparities between MetS-afflicted individuals and their healthy counterparts, they falter in explicitly delineating the importance of specific factors. The application of feature importance and Cox risk ratio models in the XGBoost framework provides the nuanced ability to differentiate between key factors, and by using these methods, we can quickly find nutrients that may have some impact on a disease and then analyze them further, a feat not possible with traditional statistical techniques.

The use of the NHANES dataset, a comprehensive compilation of population data, underpins our study. However, we did not analyze some known risk factors, such as smoking and physical activity, for example, because of missing data. This can lead to distorted data being analyzed; specifically, some diets that may be healthy are considered unhealthy because of smoking or an extreme lack of exercise. The selection of a subset of 5838 participants necessitated the application of SMOTE to mitigate category imbalance, a technique that, while effective, may introduce biases reflecting synthetic rather than actual data distributions. This is particularly relevant in the analysis of middle-aged and elderly populations; this is because there is a large population of middle-aged and elderly people suffering from MetS, which leads to a small population of OCH (n = 162). After SMOTE treatment, the resulting population of OCH will also be limited to the original population, which may limit the accuracy and completeness of some nutritional factors. NHANES is a cross-sectional study that does not allow for a direct causal relationship between nutrition and MetS. Therefore, the further validation of the potential associations between nutrients and MetS identified above will need to be pursued through population tracking and other means.

Beyond this, we recognize that the assessment of dietary intake is a complex process in which the intake of various nutrients can amplify or mitigate their respective effects and that supplements were not considered during our experiments. XGBoost was chosen as our primary analytical tool in the model selection, and we acknowledge its strengths in handling complex datasets but also recognize the potential for alternative models or parameter configurations to produce better results. Similarly, our risk analysis utilizes a Cox model under uniform event time constraints, a simplification that may affect the estimation of time-dependent risk factors.

## 5. Conclusions

In conclusion, this study harnesses the power of advanced ML technologies to shed light on the intricate relationship between dietary intake and MetS, underlining the critical need for age-specific dietary recommendations within public health initiatives. Our research not only lays the groundwork for subsequent inquiries but also paves the way for the formulation of precise public health policies, thus making a notable contribution to the discipline.

## Figures and Tables

**Figure A1:**
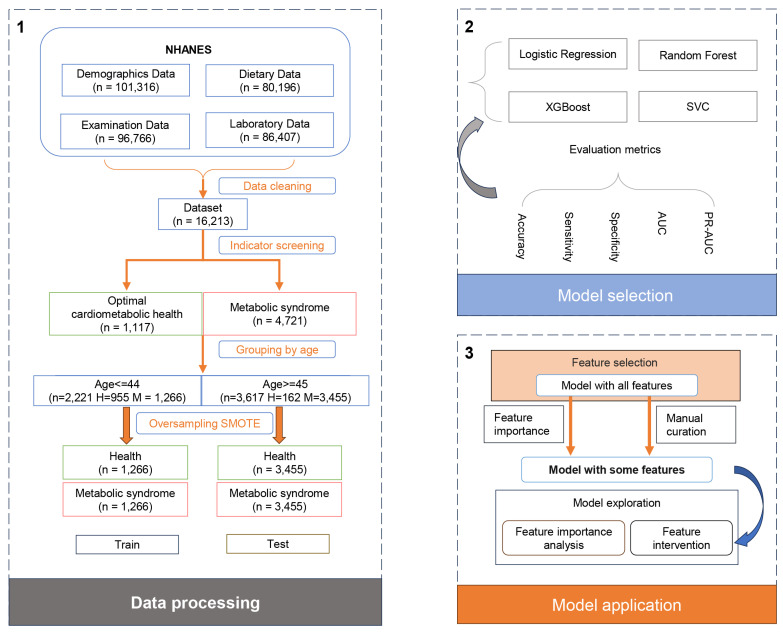
Approach overview. This figure outlines the sequential execution steps of our methodology. Step 1 involves data processing, detailing the handling of temporary dataset 1. In Step 2, we assess four models against five evaluation metrics to determine the most suitable model for selection. Step 3 focuses on feature selection within the dataset, applying feature importance and manual examination to construct a new model. This model undergoes further analysis for feature importance and the impact of feature adjustments.

## Data Availability

The dataset can be accessed through https://www.cdc.gov/nchs/nhanes/index.htm (accessed on 16 January 2024). Anonymized data may become available to third parties after request to the email address caichengl@163.com.

## Appendix A

**Table A1:** The physical examination metric and nutritional indicator analysis of 1117 in optimal cardiometabolic health and 4721 with a metabolic syndrome.

Characteristics	Optimal Cardiometabolic Health	Metabolic Syndrome
N	1117	4721
Sex (men%)	26.41%	47.11%
Age	30.79 ± 12.79	55.67 ± 16.58
Weight (kg)	59.36 ± 8.8	90.35 ± 20.9
Height (cm)	166.07 ± 8.89	166.76 ± 10.26
Upper Arm Length	35.62 ± 2.34	37.67 ± 2.82
Arm Circumference	27.29 ± 2.67	35.31 ± 4.79
Waist Circumference	77.2 ± 6.23	108.56 ± 13.82
Systolic (first reading)	105.41 ± 7.51	132.92 ± 18.74
Diastolic (first reading)	62.91 ± 7.87	72.86 ± 12.51
Systolic (second reading)	105.09 ± 7.44	131.56 ± 18.24
Diastolic (second reading)	62.93 ± 8.12	72.13 ± 12.71
Systolic (third reading)	104.76 ± 7.5	130.53 ± 18.1
Diastolic (third reading)	62.91 ± 8.56	71.75 ± 13.03
Direct HDL-Cholesterol (mg/dL)	64.1 ± 13.39	46.03 ± 13.17
Fasting Glucose (mg/dL)	89.68 ± 6.13	117.64 ± 28.93
Triglycerides (mg/dL)	69.38 ± 31.38	177.68 ± 98.02
Glycohemoglobin (%)	5.14 ± 0.28	5.98 ± 0.92
Total Cholesterol (mg/dL)	169.2 ± 30.79	197.39 ± 42.86
BMI	21.45 ± 2.04	32.36 ± 6.32
Energy (kcal)	2007.28 ± 766.01	1896.51 ± 721
Protein (gm)	76.93 ± 32.44	74.67 ± 30.9
Carbohydrate (gm)	250.6 ± 101.1	232.49 ± 94.69
Total fat (gm)	74.96 ± 35.03	72.86 ± 33.92
Total saturated fatty acids (gm)	24.45 ± 12.78	23.59 ± 11.97
Total monounsaturated fatty acids (gm)	27.01 ± 13.55	26.27 ± 12.98
Total polyunsaturated fatty acids (gm)	16.78 ± 8.62	16.27 ± 8.7
Cholesterol (mg)	255.63 ± 165.17	278.21 ± 174.62
Dietary fiber (gm)	15.67 ± 8.13	15.6 ± 8.02
Vitamin B1 (mg)	1.55 ± 0.71	1.47 ± 0.64
Vitamin B2 (mg)	1.96 ± 0.93	1.89 ± 0.86
Niacin (mg)	23.73 ± 10.76	22.18 ± 10.02
Vitamin B6 (mg)	1.9 ± 1	1.8 ± 0.91
Total folate (mcg)	388.58 ± 192.74	360.68 ± 174.72
Vitamin B12 (mcg)	4.5 ± 3.07	4.39 ± 3.06
Vitamin C (mg)	83.33 ± 70.11	75.41 ± 65.03
Calcium (mg)	901.1 ± 467.3	828.81 ± 428.32
Phosphorus (mg)	1272.32 ± 515.23	1221.58 ± 495
Magnesium (mg)	273.16 ± 115.77	263.04 ± 107.17
Iron (mg)	14.29 ± 6.93	13.71 ± 6.54
Zinc (mg)	10.64 ± 5.29	10.4 ± 5.15
Copper (mg)	1.19 ± 0.55	1.11 ± 0.48
Sodium (mg)	3315.06 ± 1430.18	3158.27 ± 1365.69
Potassium (mg)	2426.03 ± 982.38	2440.12 ± 949.01
Selenium (mcg)	105.75 ± 47.19	103.21 ± 44.84
Caffeine (mg)	110.87 ± 132.83	145.71 ± 149.88
Theobromine (mg)	33.7 ± 52.28	28.08 ± 46.79
Alcohol (gm)	6.94 ± 15.77	5.28 ± 13.97
Butanoic (gm)	0.52 ± 0.42	0.45 ± 0.37
Hexanoic (gm)	0.29 ± 0.24	0.26 ± 0.22
Octanoic (gm)	0.24 ± 0.19	0.21 ± 0.17
Decanoic (gm)	0.44 ± 0.33	0.4 ± 0.3
Dodecanoic (gm)	0.72 ± 0.72	0.67 ± 0.69
Tetradecanoic (gm)	2.08 ± 1.42	1.92 ± 1.31
Hexadecanoic (gm)	13.27 ± 6.72	12.95 ± 6.35
Octadecanoic (gm)	6.06 ± 3.25	5.94 ± 3.08
Hexadecenoic (gm)	1.09 ± 0.68	1.09 ± 0.64
Octadecenoic (gm)	25.14 ± 12.75	24.44 ± 12.15
Eicosenoic (gm)	0.25 ± 0.17	0.25 ± 0.17
Docosenoic (gm)	0.02 ± 0.04	0.02 ± 0.05
Octadecadienoic (gm)	14.82 ± 7.71	14.35 ± 7.8
Octadecatrienoic (gm)	1.51 ± 0.87	1.49 ± 0.87
Octadecatetraenoic (gm)	0.01 ± 0.02	0.01 ± 0.02
Eicosatetraenoic (gm)	0.13 ± 0.09	0.14 ± 0.1
Eicosapentaenoic (gm)	0.03 ± 0.06	0.03 ± 0.06
Docosapentaenoic (gm)	0.02 ± 0.02	0.02 ± 0.02
Docosahexaenoic (gm)	0.06 ± 0.1	0.06 ± 0.09
Total sugars (gm)	110.24 ± 61.83	102.55 ± 58.97
Vitamin E as alpha-tocopherol (mg)	7.52 ± 4.41	6.9 ± 3.99
Retinol (mcg)	384.31 ± 274.36	369.12 ± 270.51
Vitamin A, RAE (mcg)	565.87 ± 362	535.65 ± 347.02
Alpha-carotene (mcg)	372.35 ± 628.52	338.48 ± 589.78
Beta-carotene (mcg)	1952.63 ± 2435.91	1786.77 ± 2199.68
Beta-cryptoxanthin (mcg)	83.65 ± 130.46	88.74 ± 135.4
Lycopene (mcg)	5408.43 ± 6878.02	4747.59 ± 6305.36
Lutein + zeaxanthin (mcg)	1289.21 ± 1675.67	1242.74 ± 1651.25
Folic acid (mcg)	190.04 ± 143.47	166.34 ± 129.35
Food folate (mcg)	198.39 ± 105.13	194.45 ± 96.09
Folate, DFE (mcg)	521.51 ± 282.57	477.06 ± 255.61
Vitamin K (mcg)	95.55 ± 90.27	89.91 ± 89.54

**Table A2:** Analysis of physical examination metrics and nutritional indicators of youth cohorts (SMOTE).

Characteristics	Optimal Cardiometabolic Health	Metabolic Syndrome
N	1266	1266
Sex (men %)	25.28%	50.87%
Age	26.74 ± 7.38	33.58 ± 7.64
Weight (kg)	59.15 ± 8.46	97.81 ± 22.55
Height (cm)	166.03 ± 8.56	168.95 ± 10.11
Energy (kcal)	2007.24 ± 749.26	2134.5 ± 789.76
Protein (gm)	76.67 ± 31.71	82.12 ± 34.3
Carbohydrate (gm)	252.19 ± 98.24	265.16 ± 105.83
Cholesterol (mg)	252.33 ± 157.77	295.16 ± 184.82
Dietary fiber (gm)	15.47 ± 7.72	15.55 ± 8.46
Vitamin B1 (mg)	1.56 ± 0.7	1.56 ± 0.71
Vitamin B2 (mg)	1.92 ± 0.92	1.91 ± 0.95
Vitamin B12 (mcg)	4.41 ± 2.94	4.58 ± 3.17
Vitamin C (mg)	84.03 ± 68.84	73.25 ± 67.78
Caffeine (mg)	95.4 ± 113.28	128.48 ± 143.28
Theobromine (mg)	32.94 ± 48.9	28.27 ± 48.29
Alcohol (gm)	6.47 ± 14.88	6.15 ± 15.45
Docosenoic (gm)	0.02 ± 0.04	0.03 ± 0.05
Octadecatetraenoic (gm)	0.01 ± 0.02	0.01 ± 0.02
Eicosatetraenoic (gm)	0.13 ± 0.09	0.15 ± 0.1
Docosahexaenoic (gm)	0.06 ± 0.1	0.05 ± 0.09
Total sugars (gm)	110.14 ± 58.69	120.73 ± 69.27
Vitamin E as alpha-tocopherol (mg)	7.41 ± 4.23	7.26 ± 4.27
Retinol (mcg)	374.91 ± 261.69	356.94 ± 264.47
Alpha-carotene (mcg)	361.25 ± 584.17	294.8 ± 594.05
Beta-cryptoxanthin (mcg)	84.42 ± 125.49	75.86 ± 130
Lycopene (mcg)	5559.89 ± 6875.51	5446.79 ± 6597.58
Lutein + zeaxanthin (mcg)	1193.56 ± 1474.42	1048.66 ± 1335.73

**Table A3:** Analysis of physical examination metrics and nutritional indicators in middle-aged and elderly cohorts (SMOTE).

Characteristics	Optimal Cardiometabolic Health	Metabolic Syndrome
N	3455	3455
Sex (men %)	23.27%	45.73%
Age	55.25 ± 8.09	63.77 ± 10.49
Weight (kg)	59.24 ± 6.82	87.62 ± 19.57
Height (cm)	165.01 ± 6.8	165.96 ± 10.19
Energy (kcal)	1790.95 ± 531.69	1809.31 ± 673.49
Protein (gm)	70.19 ± 23.02	71.94 ± 29.09
Carbohydrate (gm)	213.48 ± 64.91	220.52 ± 87.27
Cholesterol (mg)	249.82 ± 135.42	272 ± 170.34
Dietary fiber (gm)	15.28 ± 6.52	15.62 ± 7.86
Vitamin B1 (mg)	1.39 ± 0.51	1.44 ± 0.61
Vitamin B2 (mg)	1.99 ± 0.64	1.88 ± 0.83
Vitamin B12 (mcg)	4.42 ± 2.53	4.32 ± 3.01
Vitamin C (mg)	77.33 ± 52.86	76.2 ± 63.99
Caffeine (mg)	195.51 ± 138.81	152.03 ± 151.77
Theobromine (mg)	37.65 ± 44.36	28.01 ± 46.23
Alcohol (gm)	8.87 ± 13.21	4.96 ± 13.37
Docosenoic (gm)	0.03 ± 0.04	0.02 ± 0.05
Octadecatetraenoic (gm)	0.01 ± 0.02	0.01 ± 0.02
Eicosatetraenoic (gm)	0.12 ± 0.07	0.14 ± 0.1
Docosahexaenoic (gm)	0.06 ± 0.07	0.06 ± 0.1
Total sugars (gm)	97.17 ± 42.73	95.89 ± 53.19
Vitamin E as alpha-tocopherol (mg)	7.66 ± 3.77	6.77 ± 3.87
Retinol (mcg)	406.66 ± 221.26	373.58 ± 272.59
Alpha-carotene (mcg)	357.35 ± 518.84	354.49 ± 587.49
Beta-cryptoxanthin (mcg)	84.73 ± 117.17	93.46 ± 137.05
Lycopene (mcg)	3587.05 ± 5068.25	4491.39 ± 6176.02
Lutein + zeaxanthin (mcg)	1487.48 ± 1595.76	1313.85 ± 1747.49

**Table A4:** A Cox risk analysis (age as covariate) was performed for screening the nutrition in a population <45 years of age.

Key	Coefficient	Lower_95_CI	Upper_95_CI	*p*_Value	*p*_Value2	−log *p*
Energy (kcal)	2.09	1.45	3.01	6.80 × 10^−5^	***p* < 0.005**	4.1673
Protein (gm)	1.87	1.25	2.79	0.00215	***p* < 0.005**	2.6668
Carbohydrate (gm)	1.95	1.39	2.73	0.00012	***p* < 0.005**	3.9265
Cholesterol (mg)	2.35	1.65	3.34	2.06 × 10^−6^	***p* < 0.005**	5.6871
Dietary fiber (gm)	0.7	0.48	1.04	0.07509	0.075	1.1244
Vitamin C (mg)	0.55	0.36	0.84	0.00599	**0.006**	2.2222
Caffeine (mg)	0.97	0.66	1.42	0.86598	0.866	0.0625
Theobromine (mg)	0.56	0.36	0.86	0.00796	**0.008**	2.0993
Alcohol (gm)	0.67	0.44	1.01	0.05638	0.056	1.2488
Docosenoic (gm)	1.35	0.73	2.51	0.34161	0.342	0.4665
Octadecatetraenoic (gm)	0.86	0.55	1.33	0.49389	0.494	0.3064
Eicosatetraenoic (gm)	2.5	1.82	3.44	1.95 × 10^−8^	***p* < 0.005**	7.7094
Docosahexaenoic (gm)	0.58	0.36	0.94	0.02848	0.028	1.5455
Total sugars (gm)	2.38	1.68	3.38	1.08 × 10^−6^	***p* < 0.005**	5.968
Vitamin E as alpha-tocopherol (mg)	0.67	0.44	1.02	0.05996	0.06	1.2222
Retinol (mcg)	0.77	0.52	1.14	0.18586	0.186	0.7308
Alpha-carotene (mcg)	0.29	0.16	0.52	3.40 × 10^−5^	***p* < 0.005**	4.4688
Beta-cryptoxanthin (mcg)	0.6	0.36	1.02	0.05985	0.06	1.2229
Lycopene (mcg)	0.93	0.65	1.33	0.68583	0.686	0.1638
Lutein + zeaxanthin (mcg)	0.22	0.11	0.42	7.64 × 10^−6^	***p* < 0.005**	5.117

**Table A5:** A Cox risk analysis (age as covariate) was performed for screening the nutrition in a population ≥45 years of age.

Key	Coefficient	Lower_95_CI	Upper_95_CI	*p*_Value	*p*_Value2	−log *p*
Energy (kcal)	2.51	1.94	3.25	2.89 × 10^−12^	***p* < 0.005**	11.53899
Protein (gm)	3.37	2.56	4.44	6.19 × 10^−18^	***p* < 0.005**	17.20796
Carbohydrate (gm)	3.28	2.44	4.39	2.21 × 10^−15^	***p* < 0.005**	14.65522
Cholesterol (mg)	2.23	1.76	2.83	3.38 × 10^−11^	***p* < 0.005**	10.47107
Dietary fiber (gm)	1.71	1.33	2.19	2.28 × 10^−5^	***p* < 0.005**	4.642278
Vitamin C (mg)	1.11	0.84	1.47	0.476642	0.477	0.321807
Caffeine (mg)	0.3	0.24	0.38	3.22 × 10^−22^	***p* < 0.005**	21.49154
Theobromine (mg)	0.53	0.39	0.71	2.47 × 10^−5^	***p* < 0.005**	4.607976
Alcohol (gm)	0.29	0.2	0.41	3.58 × 10^−12^	***p* < 0.005**	11.44574
Docosenoic (gm)	0.43	0.27	0.7	0.000572	***p* < 0.005**	3.242502
Octadecatetraenoic (gm)	1.63	1.19	2.22	0.002036	***p* < 0.005**	2.691272
Eicosatetraenoic (gm)	3.32	2.61	4.22	1.04 × 10^−22^	***p* < 0.005**	21.98281
Docosahexaenoic (gm)	1.29	0.95	1.76	0.098474	0.098	1.006681
Total sugars (gm)	1.25	0.95	1.65	0.116162	0.116	0.934938
Vitamin E as alpha-tocopherol (mg)	0.41	0.31	0.55	3.03 × 10^−9^	***p* < 0.005**	8.519156
Retinol (mcg)	0.25	0.18	0.35	9.27 × 10^−16^	***p* < 0.005**	15.03288
Alpha-carotene (mcg)	1.5	1.1	2.05	0.010036	0.01	1.998431
Beta-cryptoxanthin (mcg)	1.11	0.84	1.47	0.455166	0.455	0.34183
Lycopene (mcg)	2.42	1.94	3.01	5.61 × 10^−15^	***p* < 0.005**	14.25066
Lutein + zeaxanthin (mcg)	0.74	0.54	1.03	0.075194	0.075	1.123818

**Table A6:** Importance of features after XGBoost model training (Age ≤ 44).

Key	Importance
Weight	1653
Height	1241
Age	630
Retinol	529
Beta-cryptoxanthin	447
Caffeine	419
Vitamin C	405
Dietary fiber	389
Total sugars	385
Alpha-carotene	373
Docosahexaenoic	342
Lycopene	342
Lutein + zeaxanthin	335
Eicosatetraenoic	327
Vitamin E (alpha-tocopherol)	318
Theobromine	315
Cholesterol	312
Docosenoic	275
Carbohydrate	266
Protein	252
Energy	219
Alcohol	203
Octadecatetraenoic	192
Sex	104

**Table A7:** Importance of features after XGBoost model training (Age ≥ 45).

**Key**	**Importance**
Weight	932
Height	775
Age	510
Caffeine	415
Lycopene	379
Retinol	354
Alcohol	325
Theobromine	298
Docosenoic	287
Docosahexaenoic	281
Vitamin C	270
Alpha-carotene	268
Total sugars	261
Carbohydrate	258
Dietary fiber	255
Eicosatetraenoic	240
Vitamin E (alpha-tocopherol)	239
Octadecatetraenoic	231
Protein	223
Cholesterol	212
Beta-cryptoxanthin	199
Lutein + zeaxanthin	193
Energy	152
Sex	50
